# Differences in Carbon and Nitrogen Cycling Strategies and Regional Variability in Biological Soil Crust Types

**DOI:** 10.3390/ijms26093989

**Published:** 2025-04-23

**Authors:** Yue Tao, Yan Li, Yaojia Fu, Sijia She, Xinyue Wang, Lianghui Hou, Chaoqi Chen, Lanzhou Chen

**Affiliations:** Hubei Key Laboratory of Biomass-Resources Chemistry and Environmental Biotechnology, School of Resource & Environmental Sciences, Wuhan University, Wuhan 430079, China; taoyue@whu.edu.cn (Y.T.);

**Keywords:** arid and semi-arid regions, biological soil crusts, carbon–nitrogen cycling, nutrient acquisition strategies

## Abstract

Biological soil crusts (BSCs) play a pivotal role in maintaining ecosystem stability and soil fertility in arid and semi-arid regions. However, the biogeographical differences in soil functional composition between cyanobacterial BSCs (C-BSCs) and moss BSCs (M-BSCs), particularly how environmental changes affect nutrient cycling strategies and microbial community functions, remain poorly understood. In this study, we investigated BSCs across aridity gradients (semi-humid, semi-arid, and arid regions) in China, focusing on carbon and nitrogen cycling pathways, enzyme activities, and nutrient acquisition strategies. It was found that aridity and BSC type had significant effects on the functional characteristics of microorganisms. This was demonstrated by significant differences in various soil microbial activities including enzyme activities and carbon and nitrogen nutrient cycling. With increasing aridity, C-BSCs exhibited reduced carbon cycling activity but enhanced nitrogen cycling processes, whereas M-BSCs displayed diminished activity in both carbon and nitrogen cycling. These divergent strategies were linked to soil properties such as pH and organic carbon content, with C-BSCs adapting through nitrogen-related processes (e.g., *nifH*, *amoA*) and M-BSCs relying on C fixation and degradation. These findings provide novel insights into the functional gene diversity of BSCs across different regions, offering valuable references for ecological restoration in arid areas. Specifically, our study highlights the potential of BSC inoculation for carbon and nitrogen enrichment in arid regions, with implications for climate-resilient restoration practices.

## 1. Introduction

Biological soil crusts (BSCs), often referred to as the “living skin” of soils, composed of phototrophic cyanobacteria, algae, lichens, mosses, and heterotrophic bacteria and fungi, are ubiquitous in water-limited arid ecosystems [1,2]. BSCs play a critical role in sustaining fragile soil ecosystems and enhancing resilience against environmental stressors by regulating nutrient inputs, carbon sequestration, and nitrogen fixation across vast dryland areas [3,4]. They are essential for stabilizing soil surfaces, preventing erosion [4,5], fixing carbon and nitrogen to enrich nutrient-poor soils [6,7], regulating water cycles, and influencing vegetation succession, soil fauna activity, and ecosystem development [8,9,10]. The multifunctionality of BSCs play a pivotal role in maintaining ecosystem stability, particularly in arid and semi-arid regions.

In arid and semi-arid ecosystems, the microbial communities within BSCs exhibit high heterogeneity, shaped by factors such as physical structure, biomass, microclimate, soil development, and disturbance history [11]. BSC formation involves several successional stages, with cyanobacteria, lichens, and mosses contributing to varying degrees depending on developmental stages [12,13,14,15,16]. As BSCs develop, the abundance of heterocystous nitrogen-fixing cyanobacteria and other phototrophic species increases, thereby enhancing soil microenvironments [17]. Cyanobacteria, a dominant component of BSC microbial communities, are critical contributors to BSC formation and development [18,19,20,21]. Moss-dominated biological soil crusts (M-BSCs), as the climax stage of BSC succession, represent a critical component of surface landscapes in arid ecosystems. They play pivotal roles in carbon/nitrogen fixation, litter decomposition, and microhabitat amelioration [22]. Studies have demonstrated that the photosynthetic carbon sequestration efficiency of M-BSCs can be 3–5 times higher than that of algal crusts, with superior water retention and organic matter accumulation compared to early-stage crusts [23]. However, the microbial interaction networks, functional gene expression, and climate-adaptive mechanisms of BSCs remain poorly understood, hindering their application in ecological restoration strategies [24].

Nutrient inputs, particularly carbon and nitrogen, are limited yet critical in arid and semi-arid soil ecosystems [10,25,26]. In nitrogen-limited ecosystems, such as glacier forefields and Arctic tundra, atmospheric nitrogen fixation can account for more than half of total nitrogen input, with BSCs serving as a substantial nitrogen source [27]. Like the above-mentioned ecosystems, arid environments depend heavily on microbial communities for primary nutrient input and cycling. Cyanobacteria, as primary producers within BSCs, are crucial for carbon and nitrogen fixation and driving nutrient cycling processes [28]. Different phototrophic organisms support diverse bacterial communities, often leading to changes in ecosystem processes, such as improved nutrient cycling efficiency through enhanced nitrogen fixation and decomposition processes, as well as increased soil stability due to the accumulation of organic matter [28,29,30,31]. Despite this, the dynamic interactions between microbial groups and their functional roles in different BSC types, especially under varying environmental conditions, are not well understood. Previous studies have investigated these processes using a few molecular markers (e.g., *nifH*) to analyze microbial contributions to nutrient cycling [32,33]. However, studies examining functional genomic insights into microbial communities in BSCs remain limited [34].

While macro-genomic sequencing and bioinformatics have improved our understanding of relationships between genetic diversity and functional processes [35,36,37], the mechanisms driving carbon and nitrogen cycling strategies in BSCs remain poorly understood. In addition, the contribution of specific microbial taxa to carbon and nitrogen and cycling remains unclear. To address these gaps, this study aims to explore the compositional, structural, and metabolic differences in microbial communities across two distinct BSC types in arid regions of China. We hypothesize that environmental changes lead to distinct nutrient cycling strategies in BSCs, with variations in microbial functional genes linked to microbial functional processes. Specifically, we expect to observe significant differences in genes related to carbon and nitrogen metabolism between cyanobacterial BSCs (C-BSCs) and moss BSCs (M-BSCs). The objectives of this study are threefold: (1) to determine whether soil microbial functions reflect BSC successional stages under different aridity conditions; (2) to identify significant microbial groups or pathways differentiating the two BSC types; and (3) to explore whether microbial functions drive distinct carbon and nitrogen cycling strategies in different BSC types. By providing new insights into microbial processes in BSCs, this research will enhance our understanding of their ecological roles in arid ecosystems and contribute to future conservation and land management strategies.

## 2. Results

### 2.1. Soil Chemical Properties

Soil physicochemical properties varied significantly across aridity gradients and BSC types (Table 1). Soil physicochemical properties exhibited significant variations across aridity gradients and biological soil crust (BSC) types (two-way ANOVA, *p* < 0.05; Table 1). Aridity exerted strong main effects on total carbon (TC: F = 85.3, *p* < 0.001), total nitrogen (TN: F = 72.6, *p* < 0.001), and total phosphorus (TP: F = 132.1, *p* < 0.001), with values increasing from arid (Ningxia: TC = 13.50 ± 1.12 mg kg^−1^, TN = 1.08 ± 0.04 g kg^−1^) to semi-arid (Tibet: TC = 22.83 ± 0.79 mg kg^−1^, TN = 1.83 ± 0.09 g kg^−1^) and semi-humid regions (Inner Mongolia: TC = 35.83 ± 3.03 mg kg^−1^, TN = 2.33 ± 0.13 g kg^−1^). Moss crusts consistently enhanced nutrient retention compared to cyanobacterial crusts, demonstrating significant BSC-type effects (TC: F = 32.1, *p* < 0.001; TP: F = 45.3, *p* < 0.001), with moss crusts in semi-humid regions showing 2.1-fold higher TP (25.17 ± 3.31 vs. 12.05 ± 1.12 mg kg^−1^ in cyanobacterial crusts, *p* < 0.001). Interactive effects were observed for TC (F = 12.4, *p* = 0.001), TP (F = 8.91, *p* = 0.002), and available potassium (AK: F = 7.89, *p* = 0.005), where moss crusts amplified moisture-driven nutrient accumulation (e.g., AK increased by 45% in semi-humid vs. arid zones under moss crusts, *p* < 0.01). Conversely, pH declined with increasing moisture (arid: 7.83 ± 0.11; semi-humid: 6.97 ± 0.06, *p* < 0.05), yet cyanobacterial crusts maintained higher pH than moss crusts in arid regions (7.83 vs. 7.12, *p* = 0.005).

### 2.2. Variations in Carbon and Nitrogen Cycling Enzyme Activities and Functional Gene Abundance

**Figure 1:**
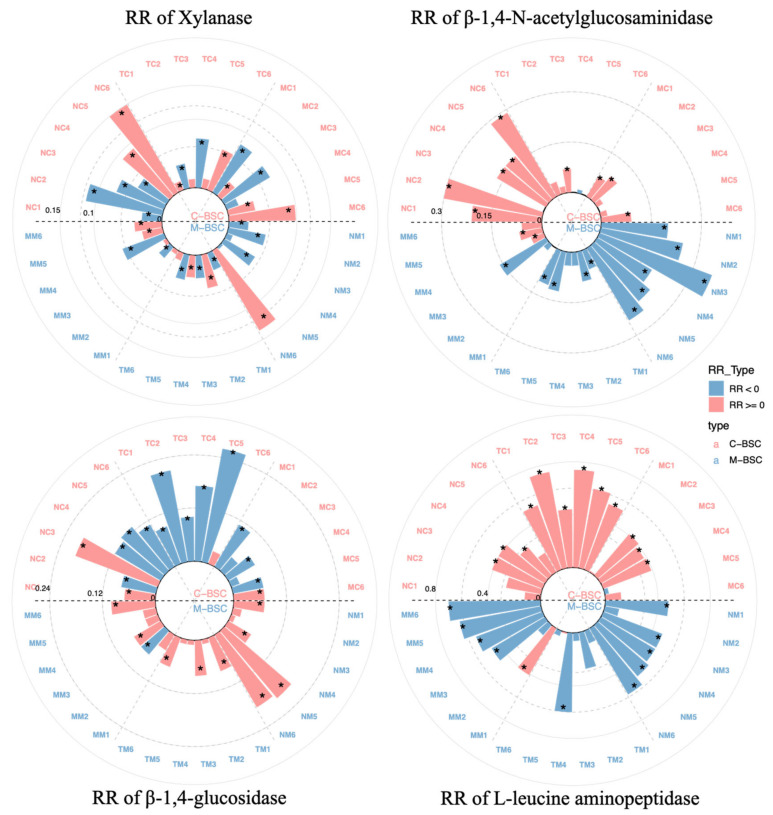
Extracellular enzyme activities of xylanase, β-1,4-N-acetylglucosaminidase, β-1,4-glucosidase and L-leucine aminopeptidase. The bar size indicates the absolute value of RR and asterisks denote significant difference between different samples (*p* < 0.05; one-way ANOVA).

The extracellular enzyme activities associated with carbon and nitrogen cycling exhibited distinct trends across aridity gradients and BSC developmental stages (Figure 1). β-1,4-Glucosidase activity decreased significantly with increasing aridity, while xylanase activity generally increased. C-cycling enzymes (β-1,4-glucosidase and xylanase) showed a marked rise in activity during BSC succession, whereas N-cycling enzymes (L-leucine aminopeptidase and β-1,4-N-acetylglucosaminidase) displayed no significant differences among stages. In M-BSCs, xylanase activity reached its peak under arid conditions (*p* < 0.05), whereas β-1,4-glucosidase activity showed no significant variation between semi-arid and arid regions (*p* > 0.05). L-leucine aminopeptidase and β-1,4-N-acetylglucosaminidase activities were notably higher in semi-humid and semi-arid regions (*p* < 0.05), with the latter enzyme demonstrating significant differences across BSC developmental stages.

**Figure 2:**
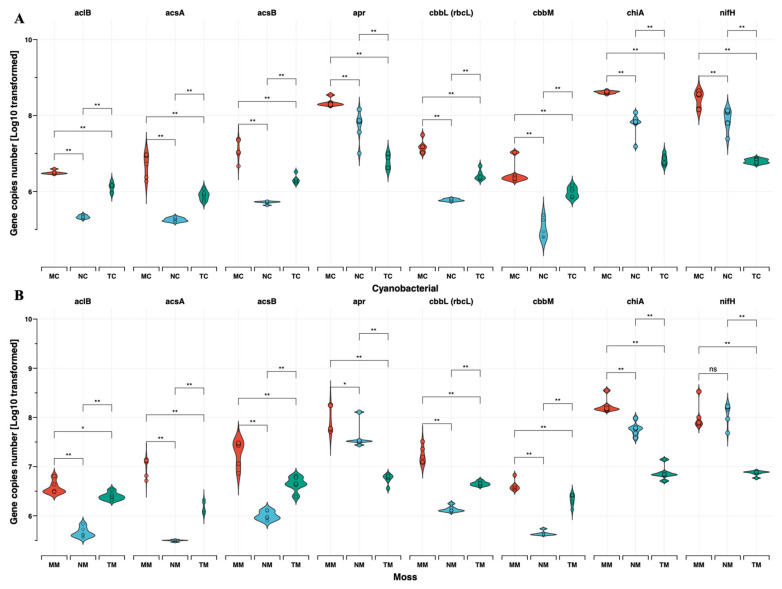
Functional gene copy numbers per gram dry weight are shown as dot plots and crossbars. (**A**) Cyanobacterial crust, (**B**) Moss crust. Crossbars are marked by the mean values and standard deviation and dots show the distribution of the single values, including N cycling function genes (a) *nifH,* (b) c*hiA* and (c) a*pr,* and carbon cycling function genes (d) *aclB*, (e) *cbbL,* (f) *cbbM,* (g) *acsA* and (h) *acsB.* (* *p* < 0.05; ** *p* < 0.01).

**Figure 3:**
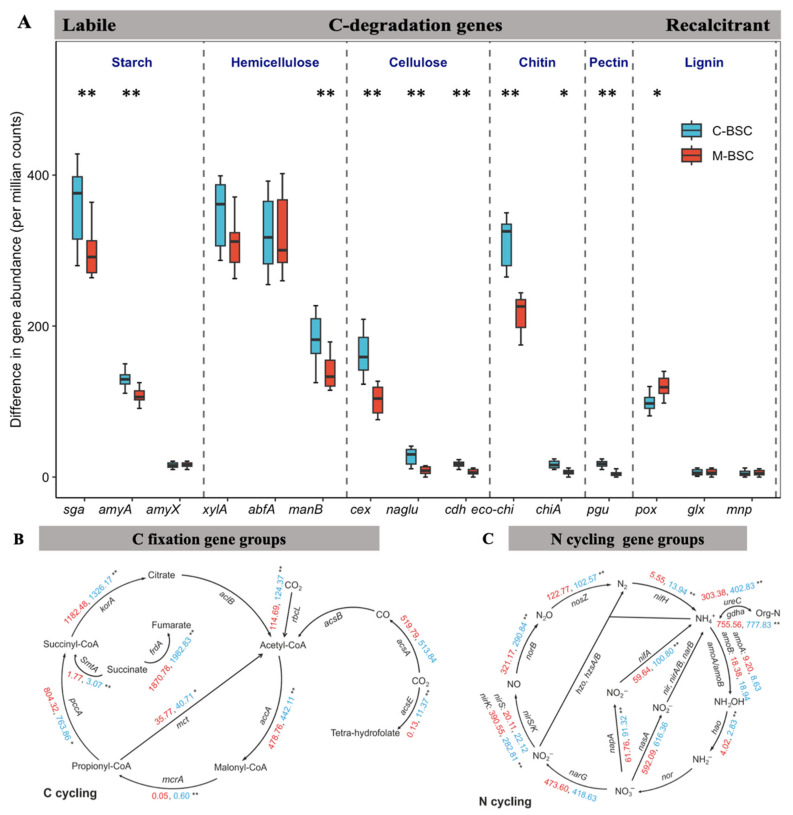
Analysis of functional gene differences between C-BSC and M-BSC. Differences in the relative abundance (counts per million–normalized) of functional genes related to C hydrolyzation (**A**), C cycling (**B**), N cycling (**C**) between C-BSC and M-BSC. The differences in the abundance of C hydrolyzed genes and genes are arranged according to the degradability of their target substrate, ranging from labile to recalcitrant. All features were significantly different between the two groups based on Wilcox sum rank test as revealed by asterisks (* *p* < 0.05; ** *p* < 0.01).

As Figure 2 shows, functional gene abundances, expressed as copy numbers per gram dry weight (copies g^−1^ dw), revealed that *nifH* (a marker for nitrogen fixation) was more abundant in semi-humid (2.08 × 10^8^ ± 1.45 × 10^8^ copies g^−1^ dw) and arid regions (9.08 × 10^7^ ± 4.96 × 10^7^ copies g^−1^ dw) compared to semi-arid zones (6.93 × 10^6^ ± 8.90 × 10^5^ copies g^−1^ dw; *p* < 0.01). Similarly, *apr* and *chiA* genes exhibited their lowest abundances in semi-arid regions (6.69 × 10^6^ ± 2.38 × 10^6^ and 8.12 × 10^6^ ± 2.36 × 10^6^ copies g^−1^ dw, respectively). Although no significant differences in C-cycling gene abundances (*cbbL*, *acsA*, *acsB*) were observed between crust types, their abundances declined with increasing aridity. Notably, moss crusts in semi-humid regions harbored the highest *cbbL* gene abundance (2.07 × 10^7^ ± 8.90 × 10^6^ copies g^−1^ dw), while cyanobacterial crusts in arid zones showed the lowest (5.85 × 10^5^ ± 4.40 × 10^4^ copies g^−1^ dw). These findings underscore the interplay between aridity, BSC succession, and microbial functional potential in shaping nutrient cycling dynamics. Figure 3A demonstrates the relative abundances of 15 genes associated with C hydrolysis that were detected and listed from labile to recalcitrant, ranked by their substrate’s biodegradability, with eight genes decomposing the labile substrates including starch, hemicellulose, cellulose, chitin, and pectin, which were higher in C-BSC than in M-BSC, while the abundances of the functional gene involved in lignin’s recalcitrant C degradation were higher in M-BSC. The *rbcL* gene, representing CO_2_ fixation ability in photosynthetic metabolism, was significantly lower in C-BSC, and similar changing patterns were found in the functional gene of *acsE* (Figure 3B). Regarding N cycling, for metabolic pathways associated with ammonia, M-BSC was associated with a significantly lower abundance of the *nifH* gene for N-fixation, and *ureC* gene for ammonification, but an increased abundance of the hao gene for nitrification, compared to C-BSC (Figure 3C).

### 2.3. Stochasticity and Determinism in Microbial Assembly of Cycling Communities

**Figure 4:**
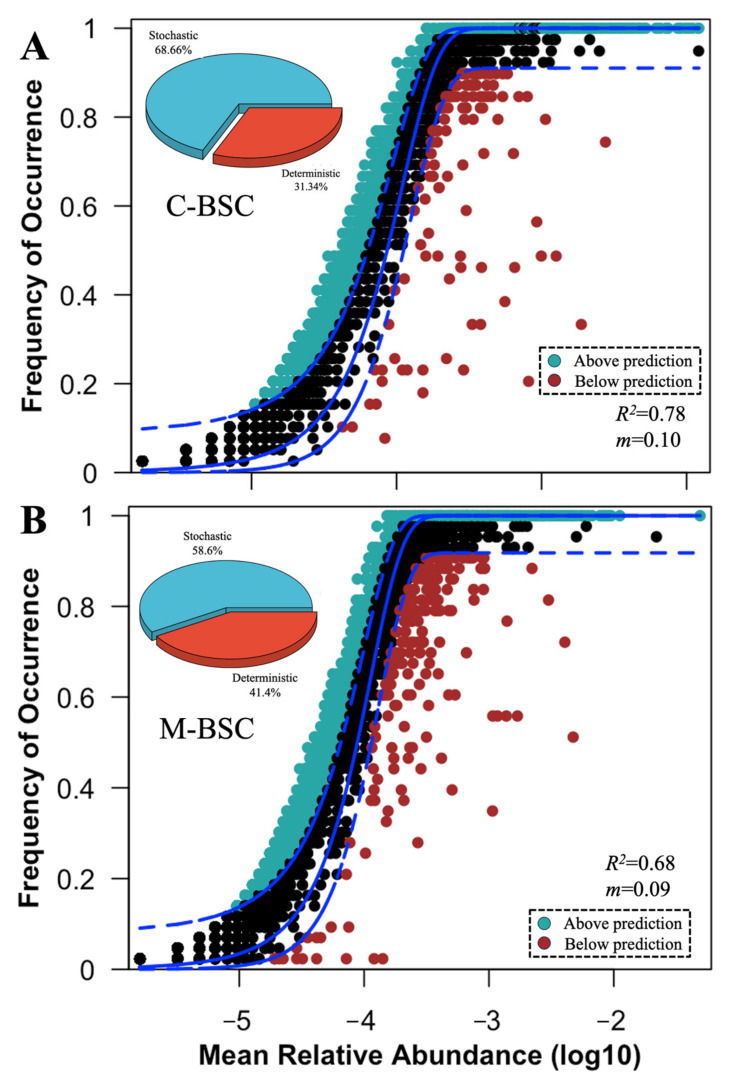
Assembly mechanisms of functional genes in C-BSC and M-BSC. The solid lines indicate the best fit to the NCM, and the dashed lines represent 95% confidence intervals of the model prediction. (**A**) Cyanobacterial crust, (**B**) Moss crust. Black dots represent the functional genes frequently of occurrence within the model predicted. Functional genes that occur more or less frequently than predicted are shown in different colors. m and R^2^ indicate the immigration rate and the fit to NCM model. Pie graphic represents the comparison of the normalized stochasticity ratio (NST) of functional gene communities in C-BSC and M-BSC.

Across all samples, considering the significant differences in carbon and nitrogen cycling, NST and NCM were employed to determine the contributions of deterministic and stochastic processes in functional gene community assembly between C-BSCs and M-BSCs (Figure 4). The NST value for functional genes with a relative importance of 58.6% and 68.7% in M-BSC and C-BSC, respectively, was beyond the boundary point (50%), indicating that the stochastic process played more critical roles in structuring the functional gene profiles of samples. We also fitted the soil functional gene assembly to the NCM and observed that the explained variation in the NCM for C-BSC (R^2^ = 0.78, Figure 4A) was higher than that for M-BSC (R^2^ = 0.68, Figure 4B). M-BSCs showed no significant differences in βNTI between nutrition cycling categories. Furthermore, deterministic processes (e.g., pH-driven selection) exerted stronger pressure on C-BSCs (mean |βNTI| = 8.46 and 6.27, respectively) than in M-BSCs (mean |βNTI| = 5.46 and 3.78).

### 2.4. Divergent Microbial Nutrient Cycling Pathways in Different Crust Types

**Figure 5:**
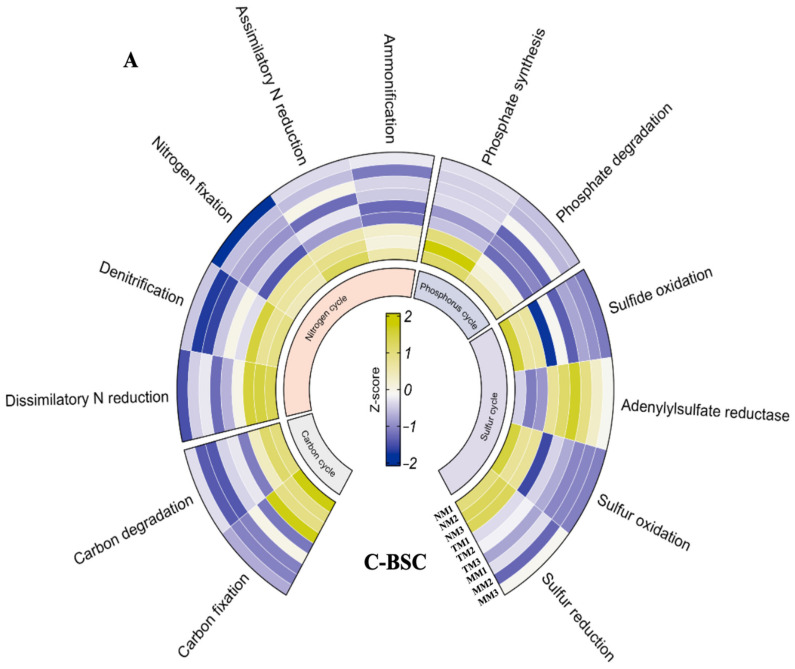

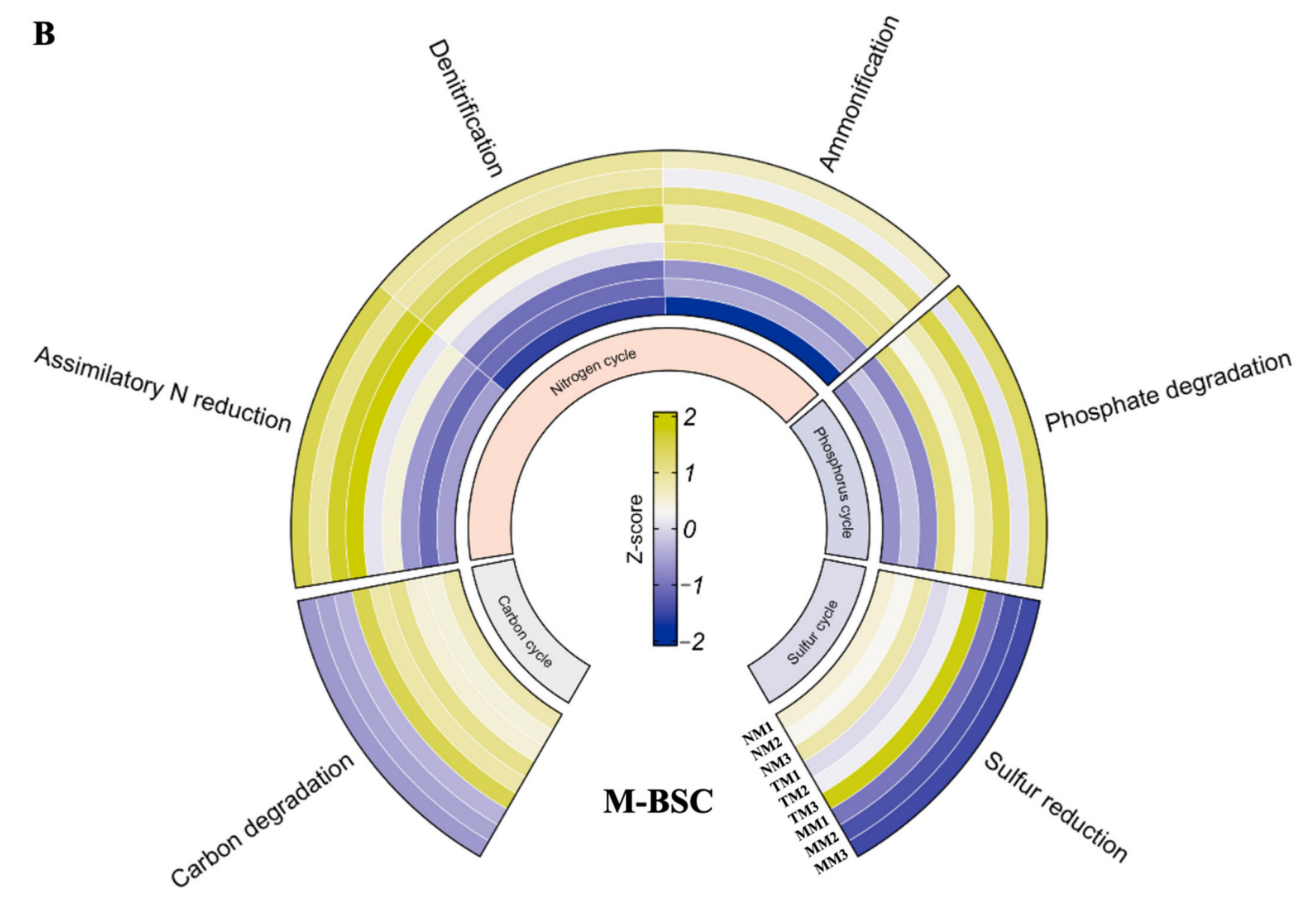
Divergent microbial metabolic pathways in nutrition cycling between cyanobacterial and moss-dominated biological soil crusts. (**A**) Cyanobacterial crust, (**B**) Moss crust.

Metabolic pathway analysis, based on KEGG database comparisons, revealed significant differences in functional gene signatures between C-BSCs and M-BSCs (Figure 5). Normalized signal intensities of genes involved in metal and non-metal biogeochemical cycles demonstrated that semi-humid M-BSCs exhibited the highest functional potential, with all detected genes showing elevated expression levels. Genes associated with carbon (C) and nitrogen (N) metabolism, including *cbbL* (RuBisCO), *nifH* (nitrogenase), and *apr* (sulfate reductase), displayed stronger signals in M-BSCs compared to C-BSCs across all regions. Notably, genes linked to C fixation (e.g., *aclB*, *acsA*) and degradation (e.g., *xylA*, *celA*) were exclusively detected in M-BSCs, whereas C-BSCs lacked C fixation genes but showed higher activity in sulfite reduction (*dsrA*), sulfur oxidation (*soxB*), and phosphate degradation (*phoD*). In contrast, M-BSCs were enriched in pathways related to C degradation (e.g., lignin peroxidase), ammonification (*gdhA*), and dissimilatory sulfite reduction (*dsrB*).

### 2.5. Key Drivers of Carbon and Nitrogen Cycling in Different Biological Soil Crust Types

**Figure 6:**
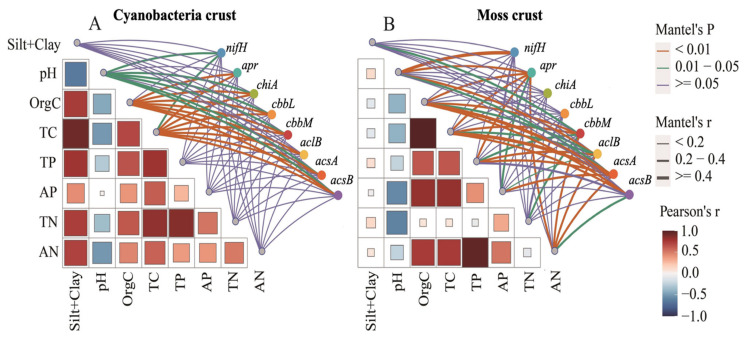
Correlations between microbial nutrient cycling functional genes and soil physicochemical properties in cyanobacterial and moss-dominated biological soil crusts. (**A**) Cyanobacterial crust, (**B**) Moss crust.

**Figure 7:**
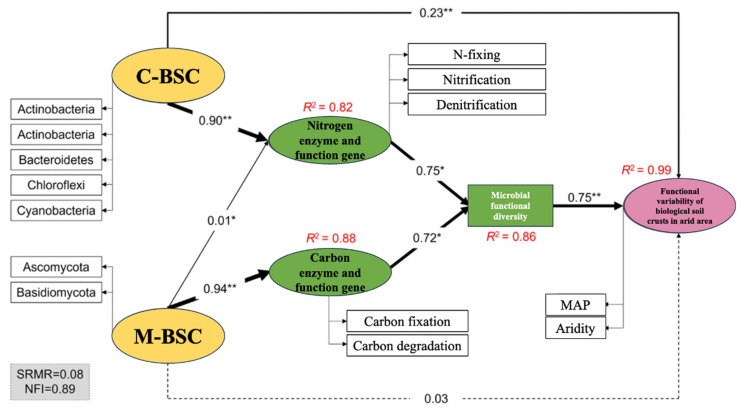
Structural equation modeling (SEM) of environmental drivers and microbial-mediated mechanisms shaping divergent nutrient acquisition strategies across aridity gradients. The width of the arrows indicates the strength of the causal relationship complemented by the standardized path coefficients (** *p* < 0.01, * *p* < 0.05). Dashed lines indicate non-significance. R^2^ indicates the explainable variance of the response variable.

Mantel tests revealed distinct environmental drivers of microbial nutrient cycling pathways in C-BSCs and M-BSCs (Figure 6). Soil pH exhibited a significant negative correlation with OrgC content (r = −0.48, *p* < 0.05), while positive correlations were observed among OrgC, TP, available AP, and AN in both crust types. Soil pH emerged as the primary limiting factor for nutrient cycling pathways across all BSCs (*p* < 0.05). Organic carbon and TC significantly influenced pathways in both crust types (*p* < 0.05), with stronger correlations in C-BSCs. In contrast, M-BSCs showed greater sensitivity to TC, AN, TN, and AP (*p* < 0.01 for TC and AN; *p* < 0.05 for TN and AP), underscoring their reliance on carbon and nitrogen availability. Structural equation modeling (SEM) further illustrated that regional environmental differences drove divergent nutrient acquisition strategies (Figure 7). For C-BSCs, microbial communities adapted to arid conditions by enhancing nitrogen fixation, nitrification, and denitrification pathways (standardized path coefficients: 0.72 to 0.85, *p* < 0.01). Conversely, M-BSCs prioritized carbon fixation and degradation processes (path coefficients: 0.64 to 0.78, *p* < 0.01), with fungal-dominated communities mediating lignin decomposition. These findings highlight the critical role of soil physicochemical properties and microbial functional guilds in shaping niche-specific nutrient cycling strategies under aridity gradients.

## 3. Discussion

Exploring the ecological characteristics of nutrient cycling under varying environmental conditions—particularly in the fragile BSC ecosystems of arid and semi-arid regions—is critical for unraveling the role of microbial communities in maintaining soil ecosystem functionality and stability. However, previous studies have predominantly focused on microbial taxonomic composition, often systematically overlooking microorganisms involved in nutrient cycling processes [38,39]. In this study, we provide strong evidence that BSC type and drought index are strongly associated with carbon and nitrogen cycling (i.e., soil nutrients, enzyme activities, and functional genes).

### 3.1. Increasing Aridity Suppresses Carbon and Nitrogen Cycling in BSCs

Our findings are consistent with global observations that aridity diminishes SOC accumulation and enzyme activities [13,40], particularly in understudied regions such as Ningxia, China. The significant decline in TC and TN with increasing aridity underscores the sensitivity of BSCs to water scarcity. These results underscore the pronounced influence of aridity and 230 BSC type on soil nutrient dynamics, with moisture availability and microbial activity acting as key drivers of biogeochemical variability. These trends reflect the dual role of BSCs in arid ecosystems: (i) their physical structure enhances soil stability, reducing erosion and creating microhabitats for microbial consortia [41], and (ii) their biological activity elevates nutrient availability (TC, TN, NO_3_^−^-N, and NH_4_^+^-N) through extracellular polysaccharide secretion and microbial biomass accumulation [42,43,44]. However, extreme aridity disrupts these mechanisms by limiting water availability and exacerbating temperature fluctuations, thereby suppressing microbial metabolism and enzyme production [45,46]. Notably, M-BSCs mitigate these pressures through superior structural integrity and higher biomass, thus providing favourable conditions for more microbial activity, which is mainly manifested in the possession of more content of soil enzyme activity-related transformed genes and biomass compared to C-BSCs [47]. This adaptive capacity explains why M-BSCs exhibited higher xylanase and β-glucosidase activities than C-BSCs in arid zones.

### 3.2. Carbon and Nitrogen Cycling Drives Adaptation in Cyanobacterial BSCs Under Arid Conditions

From semi-humid to arid regions, the genes in different regions showed different patterns of change. The copy number of C cycle-related genes decreased with the increase in aridity, while the minimum copy number of N cycle-related genes appeared in the semi-arid region. Changes in the copy number of genes associated with the C cycle are closely related to microbial biomass, while the successional development of BSC and improved environmental conditions (less aridity and more precipitation) lead to the formation of species containing more microorganisms and more complex microbial community composition. Increased microbial activity accelerates C transformation and degradation processes, which further increases biomass and promotes the development of biological soil crusts to higher successional stages and more diverse types [48,49,50]. The reason why the nitrogen cycling process is weakened in semi-arid areas may be that the BSCs in semi-arid areas face more influencing factors such as soil erosion, which makes the transition rates of ammonification, nitrification and denitrification processes not consistent, so that the functional microbial communities involved in the N transformation process cannot be formed stably even though they have higher nutrient contents and enzyme activities than those in arid areas [7,51,52].

The dominance of nitrogen-cycling genes (*nifH*, *amoA, hao*, *nirK*, and *nosZ*) in C-BSCs highlights their evolutionary specialization for nitrogen retention in water-limited environments. Cyanobacteria, as pioneer colonizers, leverage nitrogen fixation (*nifH*) to convert atmospheric N_2_ into bioavailable ammonia (NH_3_), a critical process in N-depleted arid soils [50,53,54]. Concurrently, the enrichment of nitrification (*amoA*, *hao*) and denitrification (*nirK*, *nosZ*) genes enables efficient nitrogen turnover, balancing soil NH_4_^+^-N and NO_3_^−^-N pools while minimizing gaseous N losses [55,56,57]. This metabolic flexibility allows C-BSCs to thrive under fluctuating moisture regimes, where rapid nitrogen mobilization supports microbial growth and stabilizes soil fertility [27,58]. In contrast, the reduced nitrogen-cycling potential in semi-arid regions likely stems from disrupted microbial coordination under intermediate stress, where incomplete nitrification–denitrification coupling leads to nutrient leakage [47,59]. These findings emphasize cyanobacteria as keystone engineers of nitrogen resilience in arid ecosystems, with implications for restoring degraded soils through BSC inoculation.

While aridity is a primary driver of microbial functional traits, other environmental factors significantly influence BSC dynamics. Soil pH exhibited a strong negative correlation with organic carbon content, potentially shaping microbial enzyme activities and gene expression. For instance, alkaline conditions (pH > 8.0) in arid C-BSCs favor nitrogen-fixing cyanobacteria, whereas neutral pH in M-BSCs (pH~7.0) supports fungal lignin degradation. Soil texture also plays a role: higher sand content in arid zones may reduce water retention, exacerbating moisture stress for fungi-dominated M-BSCs. Conversely, semi-humid regions with higher silt and clay content enhance nutrient retention, promoting carbon cycling in moss crusts. These findings highlight the need to integrate multiple abiotic factors (e.g., pH, texture, organic matter) into predictive models of BSC functionality under climate change.

### 3.3. Ecological Strategies of Carbon and Nitrogen Cycling Differ Between BSC Types

The divergent carbon and nitrogen-cycling strategies of C-BSCs and M-BSCs reflect niche differentiation shaped by aridity gradients. Cyanobacterial crusts prioritized nitrogen fixation and redox-driven transformations (e.g., sulfite reduction, phosphate degradation), aligning with their role as early successional pioneers in nutrient-poor environments [60,61]. In contrast, M-BSCs invested in carbon stabilization via lignin degradation and organic matter mineralization, leveraging fungal-dominated consortia to decompose complex polymers [62]. This functional divergence was mediated by soil properties: pH and organic carbon content drove pathway selection in C-BSCs, while TC, TN, and AP were pivotal in M-BSCs. Intriguingly, the weaker correlations between nitrogen-cycling genes and soil nutrients in semi-humid M-BSCs (despite high gene abundance) suggest substrate saturation or metabolic redundancy under favorable conditions [24]. Conversely, the tight coupling of *nifH* abundance and NH_4_^+^-N in arid C-BSCs (r = 0.79, *p* < 0.01) underscores nitrogen limitation as a key selective pressure. These adaptive strategies highlight the dual role of stochastic and deterministic processes in microbial assembly: while rare taxa followed environmental filtering, dominant taxa persisted via functional plasticity [35,59,63]. Although this study unifies the analytical framework through the drought gradient, local environmental differences (e.g., vegetation cover, etc.) in different cities may have an additive effect on the functioning of BSCs. Future studies need to further control for region-specific factors to more precisely resolve direct associations between drought and BSC types.

## 4. Materials and Methods

### 4.1. Study Area and Sample Collection

Sampling was conducted at 24 sites across China’s arid regions (each area dominated by 3 main cities) (latitudes 29° N–43° N, longitudes 88° E–120° E) in June to August 2021, encompassing semi-humid (AI = 0.35 to 0.60), semi-arid (AI = 0.60 to 0.85), and arid zones (AI > 0.85), where AI was the Global Aridity Index, obtained from Climate database version 2 (https://cgiarcsi.community/ 2019/01/24). The study sites ranged in elevation from 539 to 3750 m, with mean annual precipitation ranging from 227.67 to 378.00 mm and mean annual temperature ranging from 8.67 to 16.84 °C. In recent years, non-irrigated revegetation systems have been established in three cities at intervals of 1600 km, preventing infrastructure facilities from being subjected to harsh winds and sand. Multiple types of crusts form in each area due to the different development times of BSCs, and the distance between the sites in each is about 20–30 km. To account for the impacts brought by the environmental differences (except for AI) among the three cities themselves, the correlations between the dissimilarity of environmental factors and the pairwise spatial distance of sites were evaluated [46]. Environmental dissimilarity was calculated as the Gower distance using a matrix of normalized variables (Z-scores). From Supplementary Information Figure S1, it can be observed that there was a threshold effect of spatial environmental heterogeneity in three regions, with a threshold geographic distance of 500 km interval, and low environmental differences in long-distance sample pairs, suggesting that the sampling area interval did not lead to too obvious environmental differences. To minimize the impact of human disturbance, each study site was located at least 50 km away from human habitation and at least 1 km away from major roads.

At each site, four composite samples of C-BSCs and M-BSCs were collected from unvegetated areas (≥50 km from human settlements) using a sterilized shovel. The spacing between the two types of BSC distributions is between approximately 100–200 m. Each composite sample consisted of five subsamples (10 m intervals) from the top 1 cm soil layer that were homogenized and sieved (300 μm). BSCs located between sparse grass and sparse shrubs were collected with a shovel along with visibly attached subsoil and preserved in sterilised plastic Petri dishes to ensure integrity, and then samples were kept in iceboxes for transport to the laboratory. To prepare for regional signature metabolite analyses, three samples from each sample site were randomly selected and mixed thoroughly in the field to form a composite sample, so that triplicate samples were collected from each sampling area.

### 4.2. Measurement of Soil Properties

Soil pH was determined using a pH meter and soil-to-water ratio of 5:2 (*v*/*w*). Total nitrogen (N) was measured using the Kjeldahl digestion method, and exchangeable N (NH_4_^+^-N and NO_3_^−^-N) contents were analyzed by a segmented-flow autoanalyzer system after being extracted with 2 mol/L KCl (1:10 *w*/*v*). Organic carbon C was determined following the method of Wang et al. [47]. The activities of the extracellular enzyme involved in nutrient cycling were measured following the methods proposed by Kang et al. [30], including β-1,4-glucosidase (BG) and xylanase (XYL) associated with carbon cycling, L-leucine aminopeptidase (LAP) and β-1,4-N-acetylglucosaminidase (NAG) associated with nitrogen cycling. Assays were conducted using 96-well microtiter plates, with 8 replicate wells per sample per assay. Each assay included a blank, a negative control, and a quench standard, and the three controls occupied eight replicate wells each in the analysis. Next, 125 mL Tris–HCl buffer (pH = 8.0) was added to 3 g of soil for extracellular enzyme extraction. Then, an eight-channel pipettor was used to add 150 μL soil suspension liquid and 50 μL fluorometric substrate to each well of the microtiter plates. The microplates were then cultured in dark conditions at 25 °C for 4 h (NAG, XYL) and 2 h (BG and LAP) [13]. To break the reaction, a 1 mL aliquot of 1 M NaOH was added to each well. A microplate fluorometer with wavelength filters, in which excitation was 365 nm and emission were 450 nm, was used to determine fluorescence (Synergy H4 Hybrid Reader, BioTek, Winooski, VT, USA). After negative controls and quenching were corrected, the activity was expressed as nmol h^−1^ g^−1^ dry soil.

### 4.3. Real-Time Quantitative PCR

A SYBR Green^®^-based approach was applied by using a 7300 real-time qPCR machine (Applied Biosystems, Darmstadt, Germany) to quantify gene abundances according to the manufacture’s protocol. These genes were quantified to assess microbial functional potential, as they represent critical steps in carbon metabolism and fatty acid synthesis (*aclB*), acetate assimilation (*acsA*), sulfur redox cycling (*apr*), autotrophic CO_2_ fixation (*cbbL*), chitin degradation (*chiA*), and nitrogen fixation (*nifH*). The reaction mix contained 12.5 μL of SYBR Green^®^ (Thermo Fisher Scientific, Waltham, MA, USA), forward (F) and reverse (R) primers (Metabion, Planegg, Germany), 0.5 μL BSA (3%, Sigma, Darmstadt, Germany), and DEPC-treated water and was set to 25 μL. Reaction conditions, primers, calibration standards, and full names of the chosen marker genes are summarized in Table S1. To exclude inhibitory effects, dilution tests were performed prior to running qPCRs. Standard series (r^2^ > 0.99), no template controls and samples diluted to 1/64 were included in each run. To evaluate the quality of the qPCR, melting curve analyses were performed and randomly chosen samples were checked by electrophoresis on a 1.5% agarose gel. The qPCR efficiency was calculated for each primer pair with the formula Eff_slope_ = 10^(−1/slope)^−1 and was between 74 and 90% in all measurements. Values below the detection limit of 10 copies (according to manufacturer’s protocol) per reaction were set as NA.

### 4.4. DNA Extraction and Microbial Community Analysis

The analysis of soil microbial communities and functional traits in BSCs was conducted through DNA extraction, PCR amplification performed by Magigene Biotechnology Co., Ltd. (Guangzhou, China). Soil microbial DNA was extracted using the FastDNA Spin Kit (MP Biomedicals, Santa Ana, CA, USA) following the manufacturer’s protocol, with DNA concentration and purity verified via a NanoDrop One spectrophotometer (Thermo Fisher Scientific, MA, USA). Thermocycling conditions included initial denaturation at 94 °C for 30 s, annealing at 52 °C for 30 s, extension at 72 °C for 1 min (30 cycles), and a final extension at 72 °C for 10 min. Total genomic DNA of the BSC samples was extracted and subsequently analyzed by 1% agarose gel electrophoresis. The extracted DNA fragments were segmented into an average size of approximately 400 bp using the Covaris M220 (Gene Company Limited, Hong Kong, China) software (Sono Lab 7 version), in order to construct a paired terminal library. A paired-end library was constructed using NEXTFLEX Rapid DNA-Seq (Bio Scientific, Austin, TX, USA), and paired-end sequencing was performed on an Illumina Nova Seq (Illumina Inc., San Diego, CA, USA). Subsequently, low-quality reads with lengths below 50 bp, quality values below 20, or containing N bases were filtered out using the fastp software (0.23.2 version). The metagenomics data were assembled using MEGAHIT (1.2.9 version). Open reading frames (ORFs) were predicted from each assembled contig using Prodigal, and a nonredundant gene catalog was constructed using CD-HIT. The SOAP aligner was employed to align high-quality reads against the non-redundant gene catalog, with gene abundance calculated at a 95% identification rate.

Functional genes associated with carbon cycling (*cbbL*, *acsA*, *acsB*) and nitrogen cycling (*nifH*, *apr*, *chiA*) were quantified and normalized to assess their relative abundances. This comprehensive approach ensured robust characterization of microbial community structure and metabolic potential, adhering to standardized protocols for soil metagenomic analyses. The functional annotation of genes was conducted to understand the potential functions of the microbiome in different biocrusts using two databases, namely, the eggNOG and KEGG databases. Orthologous groups of proteins (COG) in the annotated representative sequences were determined by BLASTP against the evolutionary genealogy of genes and the non-supervised orthologous group (eggNOG) database using an e-value cut-off of 1 × 10^−5^. The representative sequences were also annotated in comparison to the Kyoto Encyclopaedia of Genes and Genomes (KEGG) using BLASTP (Version 2.2.28þ) with an e-value cut-off of 1 × 10^−5^. The KEGG module is a collection of manually defined functional units that is used for the annotation and biological interpretation of sequenced genomes. All sequence data were deposited into the NCBI Short Read Archive (SRA) database under accession numbers of PRJNA1246993.

### 4.5. Statistical Analyses

Data analysis was performed in R version 3.6.1 (R Core Team 2019). Data visualisation was carried out through the “ggplot2” (version 3.2.0) package [64]. We calculated Pearson coefficients and carried out Mantel tests for determining the links between environmental variables, like soil properties, and the relative abundance of microbial functional genes, to investigate variances caused by the region (semi-humid, semi-arid, arid) and sample type (cyanobacterial crust, moss crust) using the “vegan” (version 2.5-6) and “linkET” (version 0.0.3) package [65]. The deterministic and stochastic processes governing the assembly of C-BSCs and M-BSC microbial taxa were assessed using the Bray–Curtis-based Raup–Crick index (RC-Bray) and β-nearest taxon index (βNTI) values using the “picante” (version 1.8.2) package [66]. Structural equation modeling (SEM) calculated by the “piecewiseSEM” (version 2.1.0) R package [66] was used to synthesize the influences of different factors on BSC type via direct or indirect pathways. To compare soil enzyme activities’ response to different BSCs, the response ratio (RR) was calculated by using the *glht* function in the “multcomp” (version 1.4-10) package [66] for individual sites, as follows: RR = ln (X_t_) − ln (X_c_), where X_t_ and X_c_, are the means for each soil variable in the C-BSCs and M-BSCs at each site, respectively.

## 5. Conclusions

The results confirm our hypothesis that microbial assembly patterns and functional potential associated with carbon and nitrogen cycling diverge significantly during the development of BSCs under varying regional conditions. Microbial adaptive ecological strategies shift with succession according to their functional gene copy numbers related to nutrient cycling. C-BSCs in arid regions and M-BSCs in semi-humid regions exhibited the highest biogeochemical cycling potential, driven by soil nutrient availability (e.g., organic carbon, total nitrogen, and phosphorus). Soil properties—particularly pH and organic carbon content—emerged as critical drivers of microbial community assembly, functional gene expression, and metabolic pathway selection. This study demonstrates that BSC development in arid regions enhances the diversity and functional potential of N-cycling genes and pathways, with C-BSCs and M-BSCs adopting distinct strategies: cyanobacterial communities optimize nitrogen fixation, nitrification, and denitrification to cope with drought, while moss-associated consortia prioritize carbon fixation and lignin degradation. These findings establish a mechanistic link between microbial functional diversity, environmental gradients, and biogeochemical resilience, providing novel insights for the restoration of degraded dryland ecosystems through targeted BSC management.

## Figures and Tables

**Table 1:** Effects of aridity gradient and BSC type on soil properties (mean ± SE). Different lowercase letters indicate significant differences across aridity gradients (Tukey’s HSD test, *p* < 0.05). Significance codes: * *p* < 0.05, ** *p* < 0.01, *** *p* < 0.001; n.s. = not significant. TC = total carbon, TN = total nitrogen, TP = total phosphorus, AP = available phosphorus, TK = total potassium, AK = available potassium.

Parameter	Arid (Ningxia)	Semi-Arid (Tibet)	Semi-Humid (Inner Mongolia)	BSC Type Effect (C vs. M)	Interaction (Aridity × BSC)
Moisture	0.13 ± 0.02 a	0.17 ± 0.01 a	0.23 ± 0.04 a	n.s.	n.s.
pH	8.07 ± 0.53 a	7.65 ± 0.15 b	7.04 ± 0.15 b	M > C ** (F = 8.92, *p* = 0.005)	F = 4.12, *p* = 0.028
TC (g/kg)	15.62 ± 4.43 a	25.62 ± 0.79 b	35.83 ± 3.03 c	M > C *** (F = 32.1, *p* < 0.001)	F = 12.4, *p* = 0.001
TN (g/kg)	1.08 ± 0.04 a	1.83 ± 0.09 b	2.33 ± 0.13 c	M > C *** (F = 28.7, *p* < 0.001)	F = 5.21, *p* = 0.019
TC/TN	12.50 ± 0.56 a	12.49 ± 0.43 a	15.38 ± 0.91 b	n.s.	F = 4.02, *p* = 0.045
NH_4_^+^-N (mg/kg)	10.00 ± 0.57 a	10.83 ± 0.31 ab	13.17 ± 0.60 b	M > C * (F = 5.44, *p* = 0.029)	F = 4.32, *p* = 0.037
NO_3_^−^-N (mg/kg)	14.17 ± 0.34 a	16.17 ± 0.17 b	19.50 ± 0.34 c	M > C ** (F = 9.87, *p* = 0.004)	F = 6.12, *p* = 0.012
TP (g/kg)	2.33 ± 0.21 a	7.33 ± 0.42 b	25.17 ± 3.31 c	M > C *** (F = 45.3, *p* < 0.001)	F = 8.91, *p* = 0.002
AP (mg/kg)	6.79 ± 2.74 a	13.53 ± 2.42 a	29.94 ± 10.82 a	n.s.	n.s.
TK (mg/kg)	4.17 ± 0.87 a	10.83 ± 0.60 b	35.50 ± 4.50 c	M > C *** (F = 51.2, *p* < 0.001)	F = 14.7, *p* = 0.001
AK (mg/kg)	11.33 ± 0.21 a	13.17 ± 0.17 b	16.50 ± 0.56 c	M > C *** (F = 24.8, *p* < 0.001)	F = 7.89, *p* = 0.005

## Data Availability

Data will be made available on request.

## Supplementary Materials

The following supporting information can be downloaded at https://www.mdpi.com/article/10.3390/ijms26093989/s1.
