# Supplementary material for: Differences in Carbon and Nitrogen Cycling Strategies and Regional Variability in Biological Soil Crust Types
Source: Int J Mol Sci. 2025 Apr 23;26(9):3989. doi: 10.3390/ijms26093989 (PMC12071523; doi:10.3390/ijms26093989)
Supplement: Supplementary file 1 [file ijms-26-03989-s001.zip › ijms-3522958-supplementary.pdf]

Supplementary Information

Supplementary Fig.S1 Study area and sampling sites of regional. This map is free to use and could be acquired from BIGEMAP (http://www.bigemap.com) and related environmental and climate date was obtained from open source geospatial data cloud (http://www.gscloud.cn).

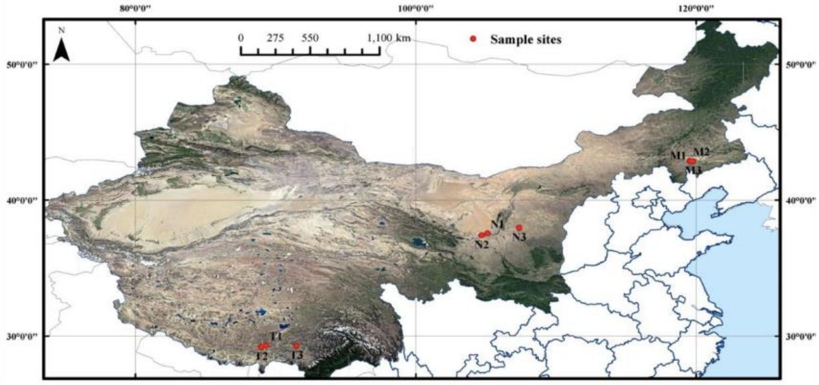

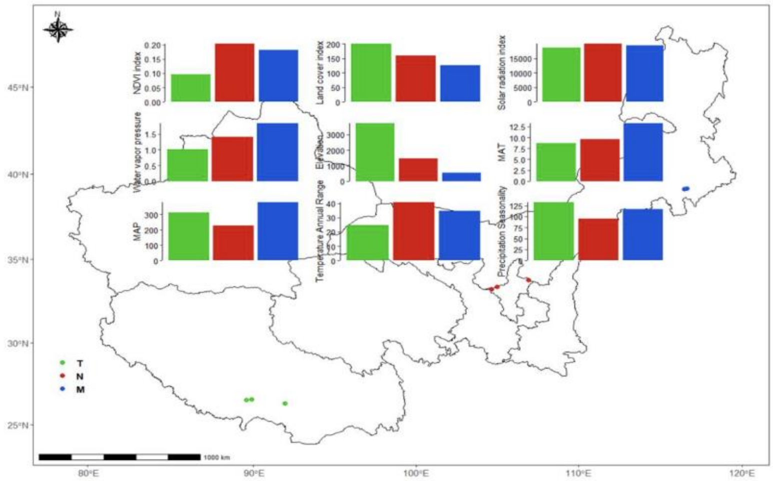

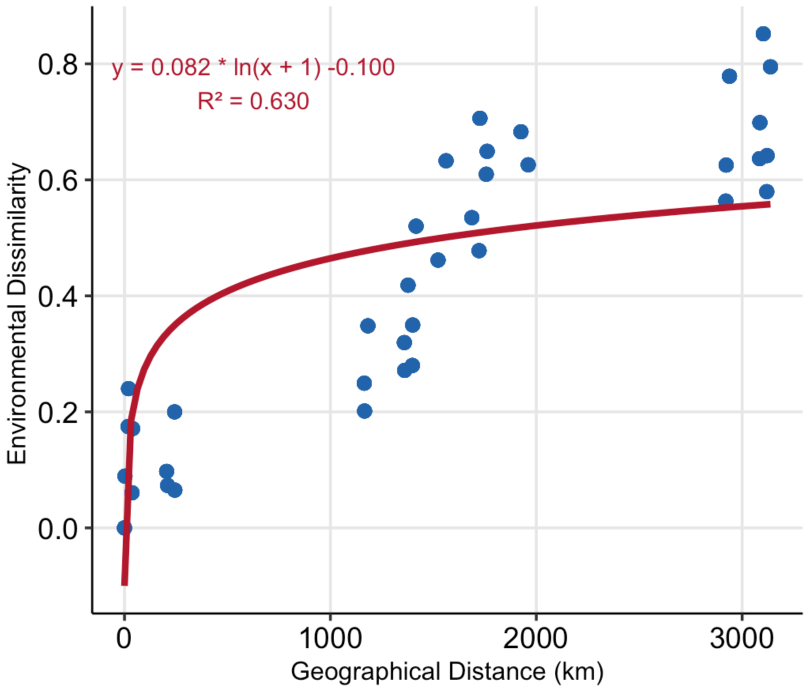

Table S1. Primers for bacteria, cyanobacteria, fungi, mosses, eukaryotic photoautotrophs, and N-cycle related genes and microbes.

Targets	Primers	Sequence (5'-3')	Annealing temperature	Reference
aclB	aclB-F	GAYGARATGGARTTYGG	55°C	Hattori et al. (2005)
	aclB-R	CCRCARTCRTGRTGRTC		
acsA	acsA-1F	TGGGACTACCTSTTCAC	58°C	Luton et al. (2002)
	acsA-2R	CGCTTCATYTGRTAGTT		
acsB	acsB-F	GARCTNGGNCARATGTAYGG	60°C	Müller et al. (2015)
	acsB-R	CCANGCRTANACRTCNAC		
apr	AprA-1-F	TAYGAYGGICAYGARGGIAC	55°C	Meyer & Kuever (2007)
	AprA-3-R	CCRTGYTGRTGRTCRTG		
cbbL(rbcL)	cbbLR-F	AAGGAGGGMATCWHTTCTACCTAA	56°C	Holmes et al. (1999)
	cbbLR-R	TCGATAGACTCCTTAGTACCACCA		
chiA	chiA-F	GACGGTATCGACAAGATGC	60°C	Williamson et al. (2000)
	chiA-R	CGCACGGTATTGTTGTAGG		
nifH	PolF	TGCGAYCCSAARGCBGACTC	55°C	Poly et al. (2001)
	PolR	ATSGCCATCATYTCRCCGGA		
	narG-R	CGTAGAAGAAGCTGGTGCTGTT		

Table S2. Variations in soil physicochemical properties and nutrient concentrations across aridity gradients and biological soil crust types. NC and NM: Cyanobacterial BSCs (C-BSCs) and moss BSCs (M-BSCs) samples collected from Ningxia，respectively；TC and TM: C-BSCs and moss BSCs samples collected from Tibet，respectively；MC and MM: C-BSCs and moss BSCs samples collected from Inner Mongolia ，respectively.

Samples	lat	lon	miosture	pH	TC	TN	TC/TN	NH4_N	NO3_N	TP	AP	TK	AK
NC1	105.10	37.58	0.12	7.54	8.26	1.18	9.53	13.50	2.72	0.37	1.94	11.37	45.06
NC2	105.10	37.58	0.16	8.38	9.25	1.20	9.36	14.55	2.75	0.38	2.57	11.52	52.30
NC3	104.70	37.43	0.14	8.70	11.53	1.28	9.63	14.78	2.98	0.39	2.96	11.79	56.68
NC4	104.70	37.43	0.10	8.77	12.86	1.34	5.76	15.01	3.01	0.40	6.92	12.00	81.29
NC5	107.37	37.98	0.13	8.78	15.27	1.37	5.50	15.03	3.07	0.45	7.23	12.33	90.12
NC6	107.37	37.98	0.12	8.54	15.89	1.48	5.34	15.15	3.16	0.49	7.36	12.39	92.84
NM1	105.10	37.58	0.16	7.98	15.92	1.63	11.52	15.28	3.76	0.49	8.03	12.43	102.87
NM2	105.10	37.58	0.14	7.82	17.19	1.67	10.39	15.63	3.78	0.50	8.06	12.64	110.55
NM3	104.70	37.43	0.12	7.48	19.08	1.75	10.17	15.65	3.96	0.54	8.27	12.68	114.58
NM4	104.70	37.43	0.13	7.84	19.36	1.76	9.99	15.72	4.26	0.57	8.72	12.77	114.88
NM5	107.37	37.98	0.13	7.45	21.13	1.88	9.58	16.16	4.31	0.59	9.50	13.29	115.02
NM6	107.37	37.98	0.12	7.57	21.74	1.97	9.35	16.18	4.46	0.61	9.89	13.33	120.74
TC1	89.32	29.32	0.18	7.61	21.91	2.00	10.88	16.32	5.59	0.63	9.89	13.42	131.49
TC2	89.32	29.32	0.18	7.69	22.12	2.09	8.83	16.38	6.33	0.63	10.76	13.66	131.56
TC3	88.97	29.21	0.17	7.79	22.42	2.09	10.47	16.59	6.85	0.66	10.88	13.77	131.68
TC4	88.97	29.21	0.16	7.83	22.77	2.12	9.32	16.61	7.51	0.68	11.86	13.99	131.78
TC5	91.48	29.31	0.16	7.91	25.01	2.20	9.24	16.91	7.87	0.69	12.78	14.37	135.46
TC6	91.48	29.31	0.15	7.77	25.15	2.21	9.50	17.06	7.87	0.69	13.04	14.39	137.40
TM1	89.32	29.32	0.18	7.57	26.39	2.22	10.45	17.13	9.44	0.71	14.18	14.53	141.57
TM2	89.32	29.32	0.19	7.40	27.26	2.37	10.28	17.15	9.46	0.71	14.39	14.66	142.27
TM3	88.97	29.21	0.16	7.47	27.44	2.38	10.74	17.41	9.63	0.71	15.15	14.84	143.11
TM4	88.97	29.21	0.19	7.68	27.59	2.40	10.76	17.49	12.09	0.73	15.50	15.05	143.65
TM5	91.48	29.31	0.17	7.60	27.91	2.42	10.96	17.57	12.42	0.75	16.12	15.18	165.20
TM6	91.48	29.31	0.18	7.51	31.41	2.56	10.75	17.81	13.67	0.77	17.82	15.29	170.68

MC1	119.53	42.89	0.23	7.04	32.73	2.62	11.05	19.16	13.92	0.80	18.96	15.45	173.51
MC2	119.53	42.89	0.21	7.04	32.95	2.68	10.91	19.20	14.26	0.82	19.29	15.58	176.20
MC3	119.56	42.88	0.26	7.07	33.95	2.72	11.02	19.38	14.86	0.85	20.15	15.81	176.36
MC4	119.56	42.88	0.16	7.07	35.51	2.84	11.11	19.49	15.44	0.87	20.23	15.83	178.43
MC5	119.77	42.88	0.21	7.17	36.11	2.89	10.90	19.84	15.62	0.87	20.24	15.84	178.93
MC6	119.77	42.88	0.24	7.36	36.95	2.95	12.01	19.88	18.14	0.88	26.49	16.16	179.56
MM1	119.53	42.89	0.25	7.15	38.48	3.03	10.58	20.01	18.45	0.90	28.75	16.26	181.61
MM2	119.53	42.89	0.26	7.02	42.86	3.08	10.51	20.56	20.20	0.91	35.06	17.79	183.28
MM3	119.56	42.88	0.22	7.08	50.68	3.54	10.74	20.71	23.71	0.93	36.84	17.80	183.99
MM4	119.56	42.88	0.25	6.81	53.35	3.67	11.74	20.80	27.04	0.94	38.62	18.08	196.52
MM5	119.77	42.88	0.20	6.91	56.95	3.76	11.50	21.37	27.59	1.00	46.80	19.70	200.94
MM6	119.77	42.88	0.30	6.80	60.49	3.88	11.27	21.78	31.12	1.03	47.91	19.71	214.50

Supplementary Table S3 Comparative analysis of the relative abundance of enzymatic functions involved in carbon fixation in C-BSCs and M-BSCs

KEGG module	Definition	M-BSCs (Mean ± SD, n=12)	C-BSCs (Mean ± SD, n=12)	F value	Sig.
M00165	Reductive pentose phosphate cycle (Calvin cycle)	0.2770 ± 0.0120	0.2733 ± 0.0125	8.8011	**0.0198**
M00167	Reductive pentose phosphate cycle, glyceraldehyde-3P => ribulose-5P	0.1924 ± 0.0125	0.1895 ± 0.0115	6.179	**0.0384**
M00172	C4-dicarboxylic acid cycle, NADP - malic enzyme type	0.1254 ± 0.0118	0.1246 ± 0.0118	0.7368	0.5863
M00171	C4-dicarboxylic acid cycle, NAD - malic enzyme type	0.1007 ± 0.0108	0.1034 ± 0.0114	17.173	**0.0028**
M00166	Reductive pentose phosphate cycle, ribulose-5P => glyceraldehyde-3P	0.0846 ± 0.0127	0.0838 ± 0.0119	0.3762	0.6864
M00169	CAM (Crassulacean acid metabolism), light	0.0838 ± 0.0115	0.0773 ± 0.0120	50.3592	**0**
M00168	CAM (Crassulacean acid metabolism), dark	0.0734 ± 0.0107	0.0789 ± 0.0104	344.61	**0**
M00170	C4-dicarboxylic acid cycle, phosphoenolpyruvate carboxykinase type	0.0626 ± 0.0113	0.0691 ± 0.0106	156.6348	**0**

Supplementary Table S4 Comparative analysis of the relative abundance of prokaryotic carbon fixation pathways in C-BSCs and M-BSCs

KEGG module	Definition	M-BSCs (Mean ± SD, n=12)	C-BSCs (Mean ± SD, n=12)	F value	Sig.
M00173	Reductive citrate cycle (Arnon-Buchanan cycle)	0.3372± 0.0115	0.3393 ± 0.0114	6.180	**0.032**
M00374	Dicarboxylate-hydroxybutyrate cycle	0.2296 ± 0.0112	0.2374 ± 0.0122	58.803	**0.000**
M00376	3-Hydroxypropionate bi-cycle	0.1930 ± 0.0118	0.1914 ± 0.0117	2.533	0.143
M00377	Reductive acetyl-CoA pathway (Wood-Ljungdahl pathway)	0.0396 ± 0.0107	0.0411 ± 0.0107	12.573	**0.005**
M00375	Hydroxypropionate-hydroxybutylate cycle	0.0995 ± 0.0115	0.0956 ± 0.0113	24.233	**0.001**
M00620	Incomplete reductive citrate cycle, acetyl-CoA => oxoglutarate	0.0847 ±0.0007	0.0805 ± 0.0111	64.287	**0.000**
M00579	Phosphate acetyltransferase-acetate kinase pathway, acetyl-CoA => acetate	0.0164 ± 0.0107	0.0148 ± 0.0107	15.413	**0.003**

Supplementary Table S5 Comparative analysis of the relative abundance of bacterial genes involved in nitrogen transformation pathways of C-BSCs and M-BSCs

KEGG module	Definition	M-BSCss (Mean ± SD, n=12)	C-BSCss (Mean ± SD, n=12)	F value	Sig.
M00530	Dissimilatory nitrate reduction: nitrate => ammonia	0.4798 ± 0.0169	0.4156 ± 0.0129	115.925	**0.000**
M00531	Assimilatory nitrate reduction: nitrate => ammonia	0.2625 ± 0.0149	0.3216 ± 0.0287	20.088	**0.001**
M00529	Denitrification: nitrate => nitrogen	0.1677 ± 0.0137	0.1502 ± 0.0120	5.495	**0.041**
M00804	Complete nitrification: ammonia => nitrite => nitrate	0.0718 ± 0.0160	0.0723 ± 0.0195	0.016	0.903
M00528	Nitrification: ammonia => nitrite	0.0141 ± 0.0134	0.0268 ± 0.0178	13.551	**0.004**
M00175	Nitrogen fixation: nitrogen => ammonia	0.0042 ± 0.0110	0.0134 ± 0.0169	10.635	**0.009**

Supplementary Table S6 Comparative analysis of the relative abundance of bacterial genes of the nitrogen cycle in C-BSCs and M-BSCs

KO	Definition	M-BSCss (Mean ± SD, n=12)	C-BSCss (Mean ± SD, n=12)	F value	Sig.
K02588	Nitrogenase iron protein(*nifH*)	0.0021 ± 0.0107	0.0060 ± 0.0131	9.188	**0.013**
K02586	Nitrogenase molybdenum-iron protein alpha chain(*nifD*)	0.0015 ± 0.0108	0.0049 ± 0.0124	10.682	**0.008**
K02591	Nitrogenase molybdenum-iron protein beta chain(*nifK*)	0.0014 ± 0.0103	0.0047 ± 0.0124	11.039	**0.008**
K10944	Methane/ammonia monooxygenase subunit A(*pmoA/amoA*)	0.0034 ± 0.0110	0.0066 ± 0.0122	10.648	**0.009**
K10945	methane/ammonia monooxygenase subunit B(*pmoB/amoB*)	0.0033 ± 0.0110	0.0048 ± 0.0121	2.500	0.145
K10946	Methane/ammonia monooxygenase subunit C (*pmoC/amoC*)	0.0101 ± 0.0122	0.0201 ± 0.0156	16.384	**0.002**
K00370	Nitrate reductase/nitrite oxidoreductase, alpha subunit(*narG*)	0.0455 ± 0.0167	0.0344 ± 0.0148	10.789	**0.008**
K00371	Nitrate reductase/nitrite oxidoreductase, beta subunit(*narH*)	0.0230 ± 0.0120	0.0189 ± 0.0110	20.101	**0.001**
K00374	Nitrate reductase gamma subunit (*narI*)	0.0106 ± 0.0115	0.0074 ± 0.0107	22.409	**0.001**
K02567	Periplasmic nitrate reductase(*napA*)	0.0192 ± 0.0138	0.0214 ± 0.0164	0.503	0.494
K02568	Cytochrome c-type protein(*napB*)	0.0031 ± 0.0106	0.0033 ± 0.0113	0.113	0.743
K00368	Nitrite reductase(*nirK*)	0.0278 ± 0.0127	0.0283 ± 0.0130	0.100	0.759
K15864	Nitrite reductase (NO-forming)/hydroxylamine reductase(*nirS*)	0.0013 ± 0.0108	0.0007 ± 0.0103	3.516	0.090
K04561	Nitric oxide reductase subunit B (*norB*)	0.0562 ± 0.0184	0.0549 ± 0.0158	0.091	0.769
K02305	Nitric oxide reductase subunit C (*norC*)	0.0015 ± 0.0106	0.0010 ± 0.0104	2.035	0.184

K00376	Nitrous-oxide reductase(*nosZ*)	0.0110 ± 0.0120	0.0057 ± 0.0112	30.395	**0.000**
K00362	Nitrite reductase (NADH) large subunit(*nirB*)	0.3218 ± 0.0193	0.2878 ± 0.0151	22.234	**0.001**
K00363	Nitrite reductase (NADH) small subunit(*nirD*)	0.0587 ± 0.0151	0.0394 ± 0.0120	75.090	**0.000**
K03385	Nitrite reductase (cytochrome c-552) (*nrfA*)	0.0633 ± 0.0161	0.0560 ± 0.0116	1.881	0.200
K15876	Cytochrome c nitrite reductase small subunit (*nrfH*)	0.0241 ± 0.0136	0.0180 ± 0.0151	5.594	**0.040**
K00367	Ferredoxin-nitrate reductase (*narB*)	0.0272 ± 0.0143	0.0533 ± 0.0166	14.018	**0.004**
K10534	Nitrate reductase (NAD(P)H) NR	0.0014 ± 0.0107	0.0003 ± 0.0101	13.102	**0.005**
K00372	Assimilatory nitrate reductase catalytic subunit (*nasA*)	0.1643± 0.0157	0.1244 ± 0.0186	89.419	**0.000**
K00360	Assimilatory nitrate reductase electron transfer subunit (*nasB*)	0.0254 ± 0.0121	0.0093 ± 0.0120	186.818	**0.000**
K00366	Ferredoxin-nitrite reductase (*nirA*)	0.0930 ± 0.0191	0.1885 ± 0.0156	168.860	**0.000**
